# Bibliometric analysis of SGLT2 inhibitor treatment for diabetic kidney disease

**DOI:** 10.1080/0886022X.2026.2667611

**Published:** 2026-05-21

**Authors:** Dan Hu, Liangmei Chen, Ruolan Mai, Tingmin Gao, Baozhang Guan

**Affiliations:** Department of Nephrology, The First Affiliated Hospital of Jinan University, Jinan University, Guangzhou, China

**Keywords:** SGLT2 inhibitors, diabetic kidney disease, bibliometric analysis, cardiorenal protection, finerenone

## Abstract

Sodium-glucose co-transporter 2 (SGLT2) inhibitors show significant renoprotective potential for diabetic kidney disease, while comprehensive bibliometric analyses in this field are scarce. This study addressed this gap *via* a systematic bibliometric analysis, retrieving 2013–2025 relevant articles from the Web of Science Core Collection and analyzing them with CiteSpace and VOSviewer. A total of 2,415 articles were included, with annual publications growing exponentially. The United States led in publication volume and citations; the University of Groningen (167 papers) was the top institution, and a mature core-scholar-led research team and collaboration network has formed. *Diabetes, Obesity and Metabolism* (163 publications) was the most prolific journal. Co-citation analysis identified 7 distinct clusters (autophagy, ketoacidosis, renal growth, finerenone, hypoxia, glomerular filtration rate, sodium-glucose cotransporter 2) and two core directions: drug action mechanisms and clinical treatment strategies. Burst keyword and keyword analyses revealed the research focus shifted from clinical trial design to cardiorenal protective mechanisms, and recently to precision disease management and combination therapies. Future research should strengthen international collaboration, explore SGLT2 inhibitor-mineralocorticoid receptor antagonist synergies, and design innovative clinical trials with region-specific endpoints.

## GRAPHICAL ABSTRACT

**Figure:**
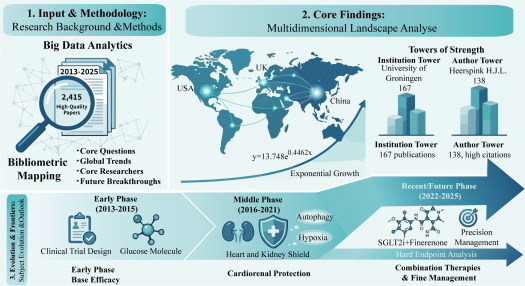

## 1. Introduction

Diabetic kidney disease is one of the most severe microvascular complications associated with diabetes and has emerged as a leading cause of end-stage renal disease globally [1]. In recent years, sodium-glucose co-transporter 2 (SGLT2) inhibitors have demonstrated significant renoprotective effects in the treatment of diabetic kidney disease, resulting in substantial growth in related research [2]. To date, seven SGLT2 inhibitors have received global approval, including the first four – empagliflozin, canagliflozin, dapagliflozin, and ertugliflozin – which are widely recognized and have been sanctioned by the U.S. Food and Drug Administration [3].

Pivotal clinical trials have provided robust evidence supporting the use of SGLT2 inhibitors in managing diabetic kidney disease and its complications. The EMPA-REG OUTCOME trial revealed that empagliflozin significantly reduced cardiovascular mortality and the risk of hospitalization for heart failure among patients with type 2 diabetes [4]. Likewise, the CANVAS program confirmed the efficacy of canagliflozin in decreasing major adverse cardiovascular events (MACE) [5]. Additionally, the DAPA-HF trial validated the cardiovascular protective effects of dapagliflozin in heart failure patients [6]. These landmark trials not only established a solid evidence base for the clinical application of SGLT2 inhibitors but also generated considerable interest in exploring their mechanisms of action and therapeutic efficacy.

Despite these achievements and expanding clinical applications, several critical issues remain unresolved. The precise mechanisms by which SGLT2 inhibitors confer broad cardiovascular protection are not yet fully understood. Proposed mechanisms include the inhibition of oxidative stress, improvements in renal hemodynamics, and modulation of metabolic pathways [7]. Furthermore, the specific targets and detailed mechanisms of action of SGLT2 inhibitors in diabetic kidney disease – particularly their effects on renal hemodynamics, oxidative stress, and endothelial function – require comprehensive investigation [8].

Given the complexity and multifaceted nature of these unresolved issues, relying solely on traditional narrative reviews or meta-analyses may be inadequate for capturing the current research landscape and future directions in this field. Bibliometric analysis, as a systematic and quantitative research methodology, offers a powerful approach to reveal research hotspots, identify emerging trends, and elucidate potential knowledge structures through meticulous extraction, analysis, and visualization of extensive literature data [9]. Unlike conventional reviews, bibliometric analysis provides a more comprehensive overview, facilitating the precise identification of research gaps and emerging directions [10]. Although research advances on SGLT2 inhibitors for treating diabetic kidney disease have been rapid, systematic bibliometric studies in this domain are notably lacking. Therefore, this study aims to address this knowledge gap through a comprehensive bibliometric analysis, focusing on three key questions: (1) How have global research trends, core countries, and research hotspots evolved in this field? (2) Which institutions, authors, and journals are the core players in this domain? (3) What are the potential future research directions and breakthroughs? By addressing these questions, this study aims to provide valuable insights for clinical practice and future research endeavors.

## 2. Materials and methods

### 2.1. Data source and search strategy

Data for this study were obtained from the Web of Science Core Collection (WoSCC) on 21 July 2025, with a focus on the SCI-EXPANDED sub-database. The search covered publications from 1 January 2013 to 30 June 2025. Since WoSCC relies on keyword-based searches in titles, abstracts, and keywords – and does not support Medical Subject Headings (MeSH) – we designed a comprehensive search strategy using relevant keywords related to diabetic kidney disease (DKD) and SGLT2 inhibitors. The search query applied was as follows: TS= ((‘diabetic nephropathy’ OR ‘diabetic kidney disease’ OR ‘DKD’ OR ‘diabetic kidney injury’ OR ‘diabetic renal disease’ OR ‘diabetic glomerulopathy’ OR (‘kidney disease’ AND ‘diabetes’) OR (‘renal disease’ AND ‘diabetes’)) AND (‘SGLT2 inhibitor’ OR ‘SGLT2 inhibitors’ OR ‘SGLT2i’ OR ‘sodium glucose cotransporter 2 inhibitor’ OR ‘sodium-glucose cotransporter-2 inhibitor’ OR ‘canagliflozin’ OR ‘dapagliflozin’ OR ‘empagliflozin’ OR ‘ertugliflozin’ OR ‘ipragliflozin’ OR ‘sotagliflozin’ OR ‘luseogliflozin’ OR ‘henagliflozin’ OR ‘bexagliflozin’ OR ‘tofogliflozin’ OR ‘remogliflozin’))

**Figure 1.**
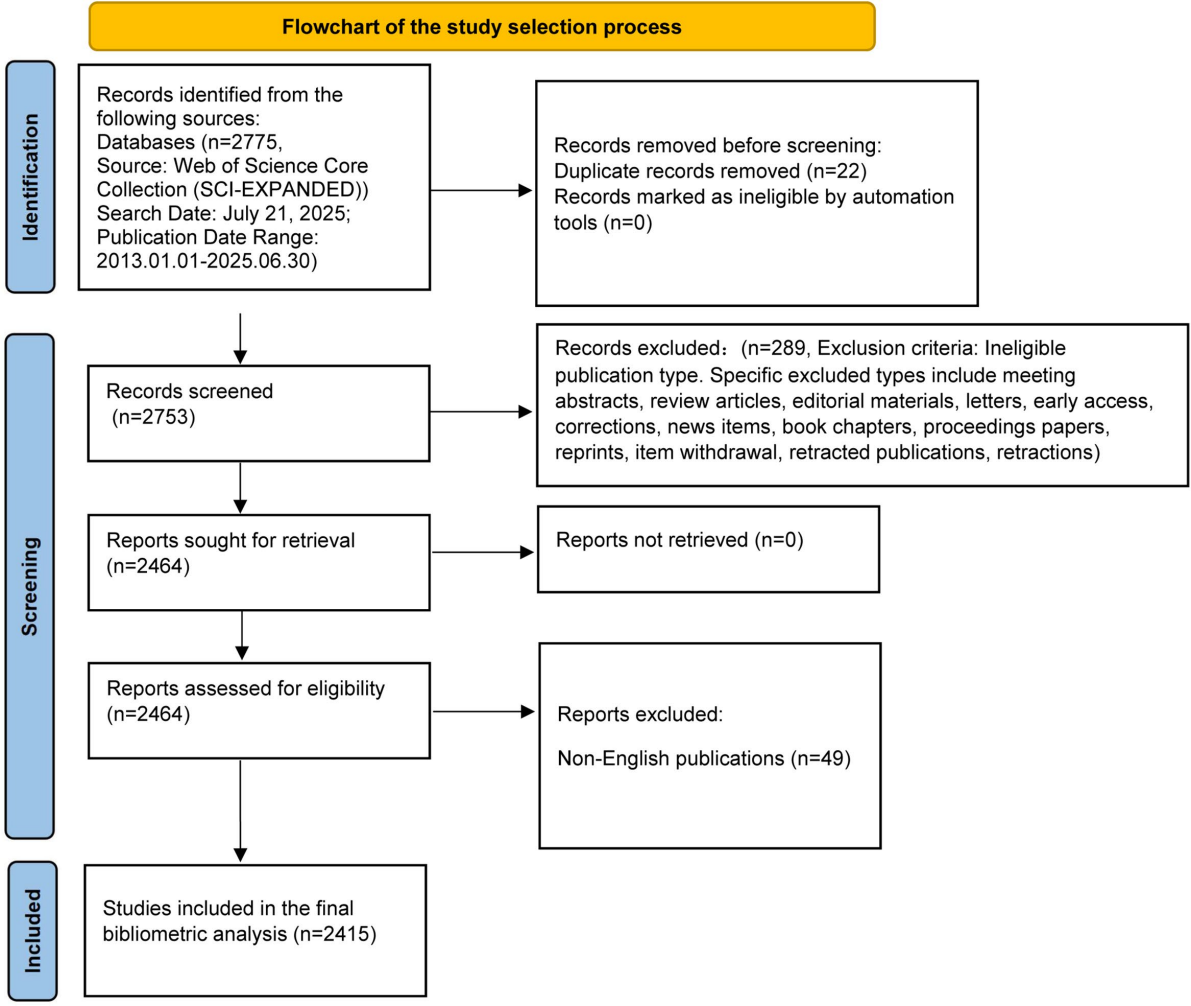
Flowchart of the study selection process.

This search returned 2,775 initial records. A systematic screening process was then applied: 22 duplicates were removed, followed by the exclusion of 289 records based on publication type (e.g., meeting abstracts, editorials), retaining only “Article’ and ‘Review’ document types. An additional 49 non-English publications were excluded, resulting in 2,415 English articles and reviews included for bibliometric analysis. To maintain citation analysis quality, only fully peer-reviewed ‘Article’ and ‘Review’ publications were considered, and the same criteria were applied to their references. The search strategy and screening criteria are summarized in a table (Supplementary Material Table S1), and the screening workflow is illustrated in Figure 1. The reporting of this study adheres to the Preferred Reporting Items for Systematic Reviews and Meta-Analyses (PRISMA) guidelines, and the checklist is provided in the supplementary files.

To comprehensively represent SGLT2 inhibitors in DKD research, all drugs in this class were included regardless of publication volume or regulatory status. Notably, certain agents such as sotagliflozin – a dual SGLT1/2 inhibitor with distinct pharmacological features [11] – were retained to ensure analytical consistency.

### 2.2. Data collection and analysis

This study adopted a bibliometric approach and primarily used two specialized software tools: VOSviewer (version 1.6.20) and CiteSpace 6.4.R2 (64-bit) Advanced. These tools enabled a systematic analysis of 2,415 publications (original articles and reviews) investigating SGLT2 inhibitors for the treatment of diabetic kidney disease. All records were retrieved from the Web of Science Core Collection. The analyses covered core bibliographic fields, including titles, authors, keywords, citation information, and the contributing countries and institutions.

Using VOSviewer, we constructed a country-level collaboration network with a minimum threshold of 10 publications per country. The network map was generated with the co-occurrence function, and clusters were identified using the clustering algorithm. The resolution parameter was set to 1.0 to improve cluster separation. A timeline view was used to illustrate changes in collaboration patterns from 2013 to 2025. Clusters were distinguished by color to facilitate interpretation and to highlight the evolving landscape of international collaboration. For the institutional collaboration network, we applied a minimum link strength of 10. Node size indicates institutional publication output, whereas link thickness reflects collaboration intensity. For the author collaboration network, we set a minimum threshold of 15 publications per author. Because the dataset included 2,415 publications and a large author pool, a lower threshold would have produced an overly dense network and reduced interpretability. In contrast, a higher threshold could have excluded influential contributors. After preliminary analyses and iterative parameter tuning, a threshold of 15 provided a balanced focus on authors with sustained, substantial output in this field and enabled clearer identification of core collaboration clusters. Author link strength was calculated from coauthored publications. Node timing corresponds to each author’s first publication in this research area, and links represent co-authorship relationships. We also refined the layout to improve readability.

CiteSpace was used to examine temporal changes in research themes. Keyword burst detection (2013–2025; minimum frequency: 5; 1-year time slice) identified the top 25 burst keywords, indicating emerging topics and shifting research priorities. Co-citation analysis (minimum co-citation frequency: 10) was performed to identify landmark references and major knowledge bases within the field. In addition, keyword co-occurrence analysis was conducted using ‘keywords’ as the unit of analysis (minimum frequency: 45), and a density map was generated. In this map, node size represents keyword frequency, and color intensity reflects co-occurrence strength, providing a concise view of core themes. All thresholds were determined through preliminary testing to preserve key information while improving network interpretability.

We also used Scimago Graphica Beta 1.0.51 to visualize publication distributions at the country and institutional levels. The maps were ranked by publication volume in descending order. Colors and labels were adjusted to maximize clarity. Taken together, these complementary analyses delineate the main contributors, evolving thematic trends, and potential future directions in research on SGLT2 inhibitors for diabetic kidney disease. The raw records can be retrieved from the Web of Science Core Collection using the search strategy described in Section 2.1. All analytical parameters are reported here to support transparency and reproducibility.

## 3. Results

### 3.1. Global publication trends

**Figure 2.**
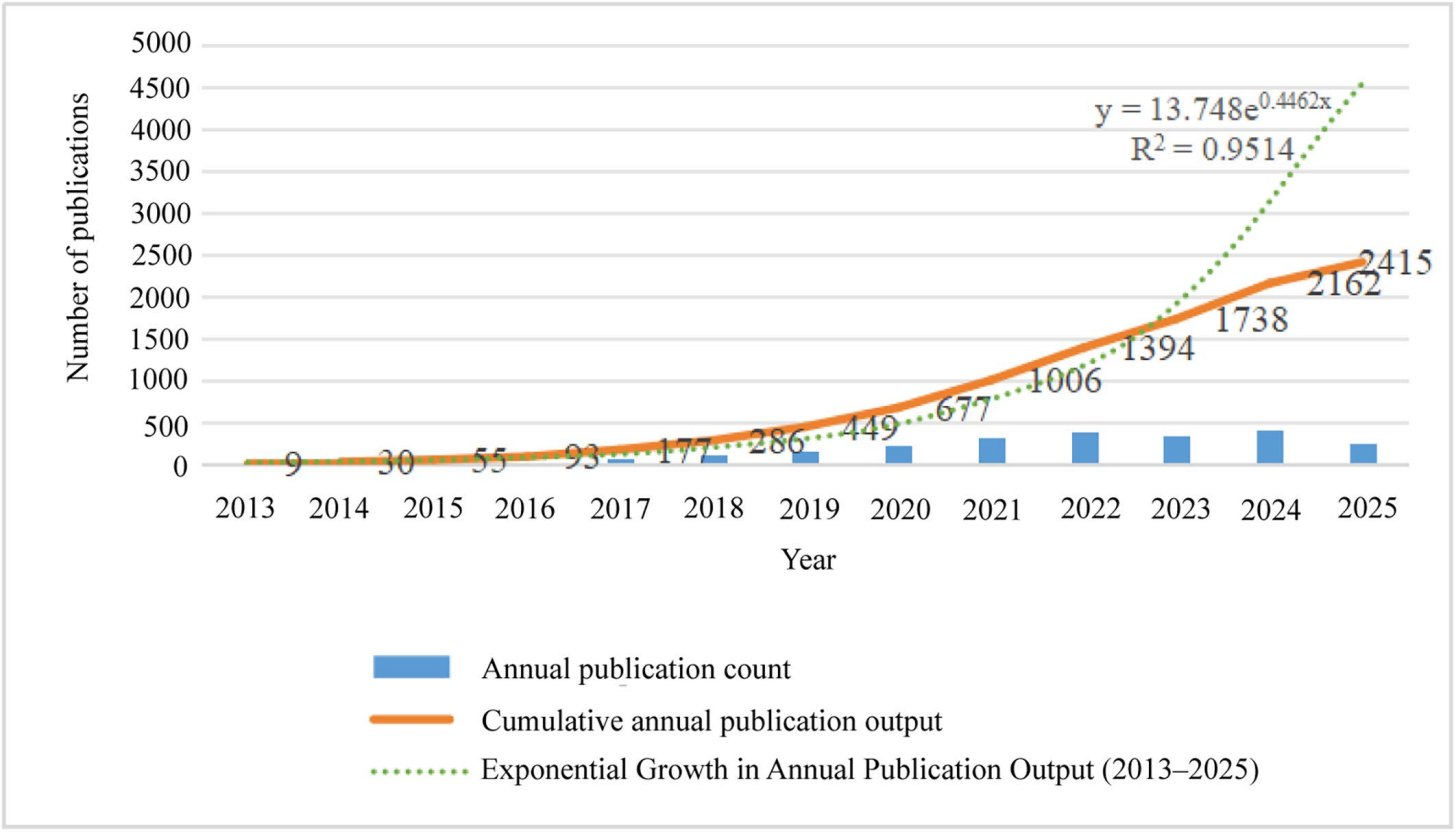
Annual number of publications on SGLT2 inhibitors for diabetic kidney disease from January 2013 to June 2025.

In international collaboration, the United States leads with 856 publications, accounting for 25.8% of the total output, while achieving a total citation count of 64,225, which represents 22.9% of the citations. China and England follow, with 482 publications (14.5%) and 326 publications (9.8%), respectively, reflecting their growing research influence in this field. Figure 2 and Table S2 illustrate the annual publication volume by country regarding the treatment of diabetic kidney disease with SGLT2 inhibitors from 2013 to 2025. Although the number of relevant studies was minimal in 2013, there has been a consistent increase in annual publications since then. Notably, from 2022 onward, the annual publication volume has stabilized at a high level and continues to increase. Concurrently, the cumulative research output has also exhibited proportional expansion, following an exponential growth model described by the equation: *y* = 13.748e^{0.4462x}, with a goodness of fit of R^2^=0.9813.

The rapid increase in publication volume is attributable to several key factors. First, the release of results from high-quality multicenter randomized controlled trials, such as EMPA-REG OUTCOME, CANVAS, and DAPA-HF, has provided essential evidence for the clinical application of SGLT2 inhibitors, thereby significantly advancing related research [12–14]. Second, the continued elucidation of the multifaceted mechanisms of SGLT2 inhibitors – particularly their cardiovascular protective, renal protective, and metabolic properties – has attracted considerable interest from researchers in various disciplines [15]. Third, the rising global prevalence of DKD and the pressing public health demand for effective therapies have further spurred research progress in this area [16].

**Figure 3.**
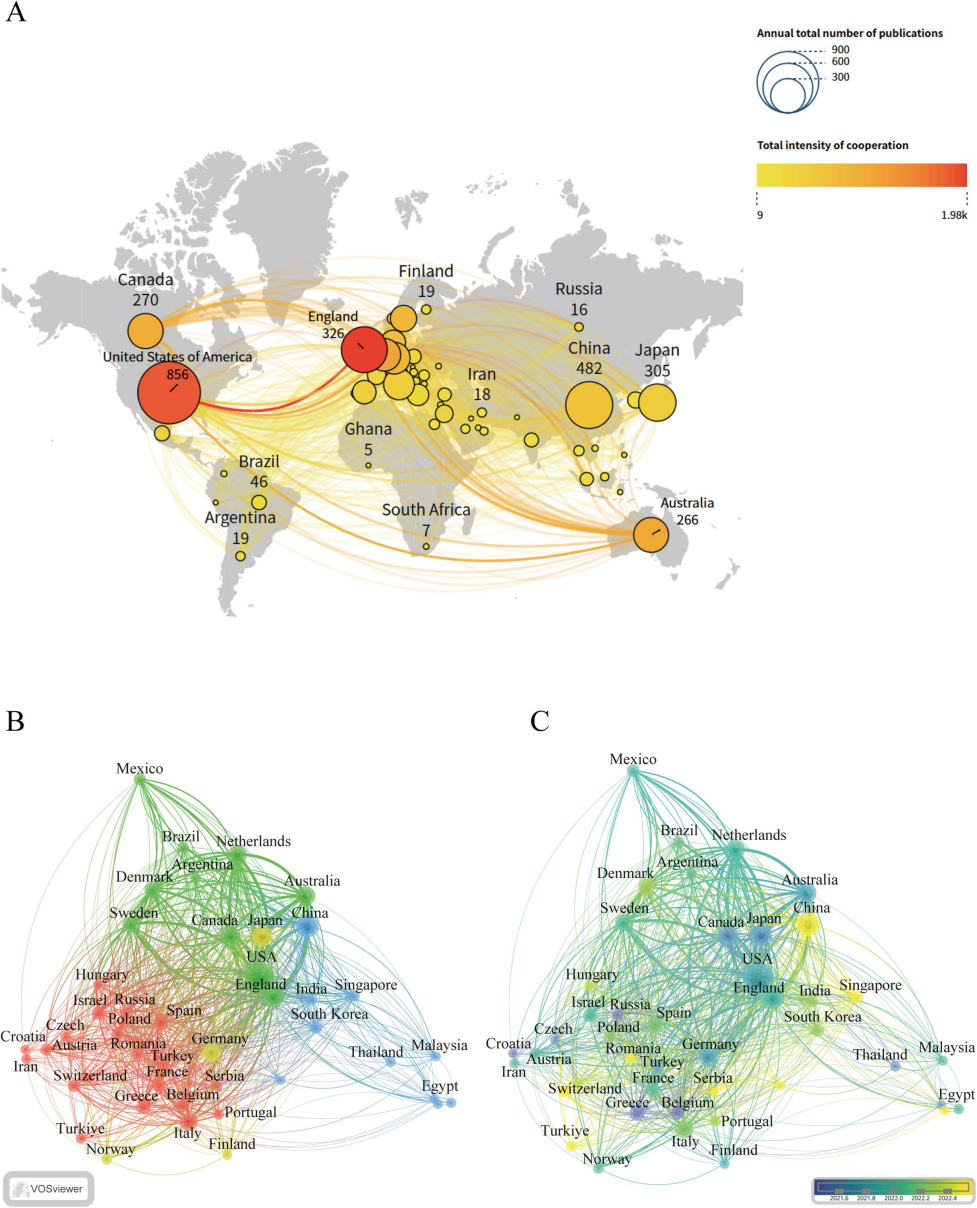
Global trends and collaboration network in research on SGLT2 inhibitors for diabetic kidney disease. A. Annual Publication Contributions by Country. This figure illustrates the annual publication volume related to SGLT2 inhibitors in the treatment of DKD from 2013 to 2025. The size of the circles indicates the number of publications, while the gradient from yellow to red represents the intensity of collaboration. The United States, China, and England ranked in the top three, highlighting their significant research investments in this field. B. International Collaboration Clusters. This figure utilizes cluster analysis to reveal the collaboration patterns among different countries. Distinct colors represent various collaborative clusters. The blue cluster includes the United States, Canada, and Australia, while the green cluster comprises China and India, both reflecting close cooperative relationships. C. International Collaboration Timeline. This figure presents the evolving dynamics of international collaboration in SGLT2 inhibitor research from 2013 to 2025. It highlights a significant surge in research activity from Japan, Canada, and Australia since 2020, alongside a rapid increase in output from China and Switzerland starting in 2021.

Analysis of the international collaboration network (Figure 3) reveals the geographic distribution and clustering patterns of research in this field. The findings indicate that the United States, China, England, and Japan are leading in publication output, forming a North American collaborative cluster centered around the United States, connecting with Canada and Australia, as well as an Asian research cluster characterized by close cooperation between China and India. Notably, while Germany, France, and Italy form a tightly-knit European collaborative group, their interaction with the North American cluster remains relatively limited. Since 2020, Japan, Canada, and Australia have significantly increased their research activity, while China and Switzerland have shown rapid growth starting in 2021. These collaboration patterns highlight the complementary strengths of each country’s research resources and clinical advantages: the United States excels in basic research and clinical trial design, while other nations contribute critical support in patient cohorts and clinical practice. The strengthening global collaboration network underscores a shared focus on the role of SGLT2 inhibitors in treating diabetic kidney disease, reflecting a trend toward resource integration.

### 3.2. Regional and institutional distribution characteristics

**Figure 4.**
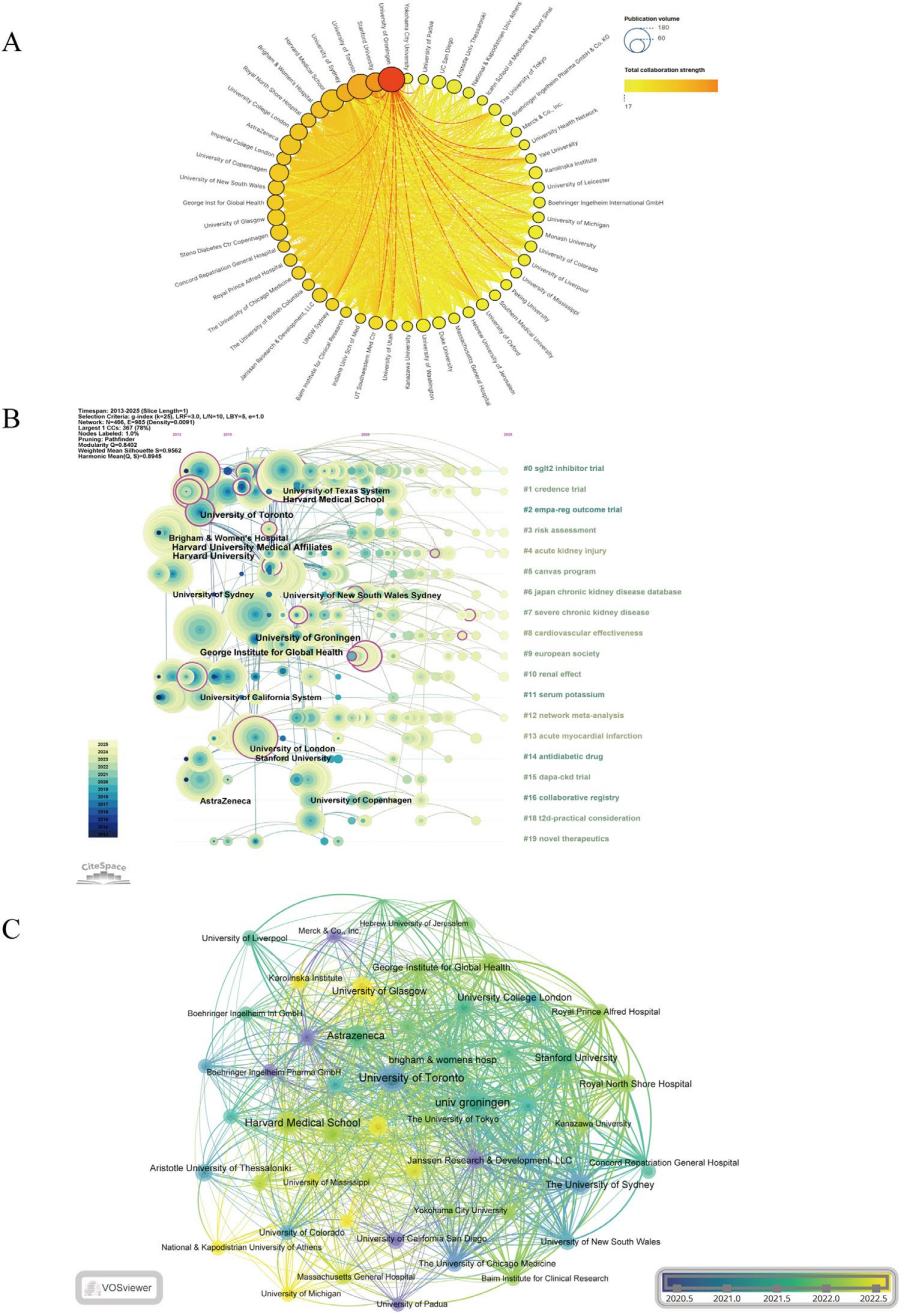
Institutional contributions to research on SGLT2 inhibitors for diabetic kidney disease. A. Institutional Collaboration Network: This figure illustrates the collaboration network among various research institutions. The size of each node corresponds to the number of publications per institution, while the thickness of connecting lines indicates the strength of collaboration. Notably, the University of Groningen and the University of Toronto occupy central positions, underscoring their extensive engagement in collaborative efforts within this field. B. Institutional Collaboration Density: The density of collaboration among institutions is vividly represented through a color gradient. Darker colors signify closer and more substantial collaboration ties. It is clear that both the University of Groningen and the University of Toronto have established strong collaborative relationships with other institutions. C. Institutional Research Timeline: This figure showcases the dynamic trends in research activities among institutions focused on SGLT2 inhibitors. It reveals a notable increase in research momentum from emerging institutions in recent years, reflecting an inherent diversification and internationalization trend within this research area.

Institutional analysis indicates that the University of Groningen is the leading institution in terms of publication outputs, with a total of 167 publications. It is followed by the University of Toronto, which has 103 publications, and Harvard University with 89 publications. For detailed information on institutional collaboration patterns, please refer to Supplementary Table S3. Figure 4(A) visually represents the collaboration network among institutions involved in SGLT2 inhibitor research. Notably, this analysis confirms the University of Groningen’s position as the top institution, with 167 publications, while the University of Toronto (165 publications) and Stanford University (94 publications) closely follow. This reflects their extensive collaborative efforts and significant influence in the field. Figure 4(B) illustrates the temporal evolution of institutional collaboration. This figure categorizes institutions based on their collaborative connections and displays the timeline of each institution’s initial involvement in cooperative activities, along with their corresponding clusters. Analysis of this figure demonstrates that institutional clusters have emerged over time, showing sustained collaborative relationships. This clearly evidences the strong ties established by the University of Groningen and the University of Toronto with other institutions. Additionally, the right side of Figure 4(B) highlights recent trends, clearly showing a dense distribution of institutional nodes across multiple clusters. This trend indicates a significant increase in the number of institutions actively engaging in the collaboration network in recent years, alongside heightened research activities across all clusters. Meanwhile, the timeline analysis in Figure 4(C) emphasizes the dynamic characteristics of research activities over time. Recently, there has been a notable rise in participation from emerging institutions within the collaboration network, in addition to the traditional core institutions. This development underscores a clear trend toward diversification in research efforts within this field and the extensive expansion of international collaboration.

### 3.3. Journal publication and co-citation analysis

Table 1 lists the top ten most active journals publishing research on SGLT2 inhibitors for diabetic kidney disease. *Diabetes, Obesity and Metabolism* ranks first, with 163 published articles accumulating 5,191 citations. It is followed by the *International Journal of Molecular Sciences* and the *Journal of Clinical Medicine*, which have published 64 and 56 articles, respectively. These findings indicate a significant concentration of research output in these specific journals. Moreover, as of 2025, most of these top ten journals have Impact Factors exceeding 3.0 and belong to the JCR Q2 category or higher. This classification further confirms their considerable authority and influence in this research field. The combination of high Impact Factors and a strong co-citation network underscores the essential contribution of these journals, providing key references for guiding future research and inspiring clinical applications.

### 3.4. Author publication and collaboration analysis

**Figure 5.**
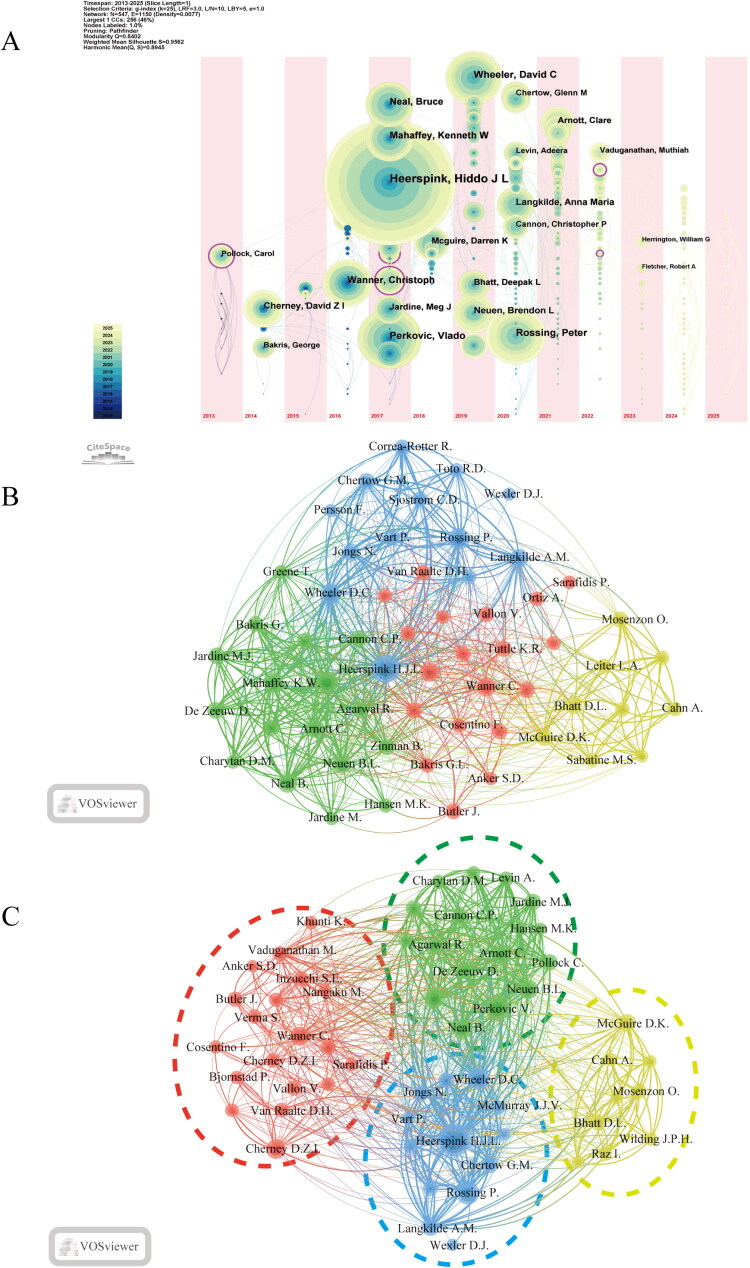
Visualization analysis of authors in SGLT2 inhibitor research for diabetic kidney disease. A. Author Output Timeline: This figure illustrates the temporal distribution of authors actively involved in research on SGLT2 inhibitors and their publication volume. Each circle represents an author, with the size indicating the number of their publications and the color shade reflecting the time of their first appearance in the dataset. B. Author Collaboration Network: This figure depicts the collaborative relationships among the authors. Each node represents an author, with the size of the node correlating to their publication volume. The thickness of the connecting lines indicates the strength of collaboration. C. Author Collaboration Clusters: This figure reveals potential collaboration patterns among authors through cluster analysis. Different colors represent distinct collaboration clusters, identifying key research teams and collaborative networks within this field.

The author analysis utilizes a timeline visualization (Figure 5(A)) to illustrate the temporal distribution of active authors in the research domain of SGLT2 inhibitors for diabetic kidney disease. Early contributors, including Carol Pollock and David Z.I. Cherney, laid a foundation of theoretical knowledge that has been essential for subsequent studies. This groundwork has facilitated a visual network analysis of author collaborations, clarifying the complex relationships among researchers. Notable scholars such as Heerspink H. J. L., Wheeler D. C., and Rossing P. have established a strong collaborative network. Among them, Heerspink H.J.L. stands out as the most influential researcher, with 138 core publications and a remarkable citation count of 13,116. These close collaborations have effectively fostered the rapid exchange and integration of academic ideas, significantly enhancing the production and dissemination of research findings (Figure 5(B)). Cluster analysis further elucidates the collaborative patterns within the author network (Figure 5(C)). The distinct collaboration clusters, indicated by different colors, clearly define the core research teams and active collaborative networks in this field. This analytical approach aids in accurately identifying key research teams and their collaboration modes, providing valuable insights for future cooperative studies.

According to Supplementary Table S4, Heerspink H.J.L. is the most prolific author in this domain, holding the highest total citation count for his research works. His high productivity reflects not only his extensive academic expertise and research depth but also his crucial role in advancing the field. Additionally, authors such as Perkovic V. and Rossing P. demonstrate significant academic influence, with their active participation in collaboration networks having a notable impact on the research landscape.

### 3.5. Co-citation analysis

**Figure 6.**
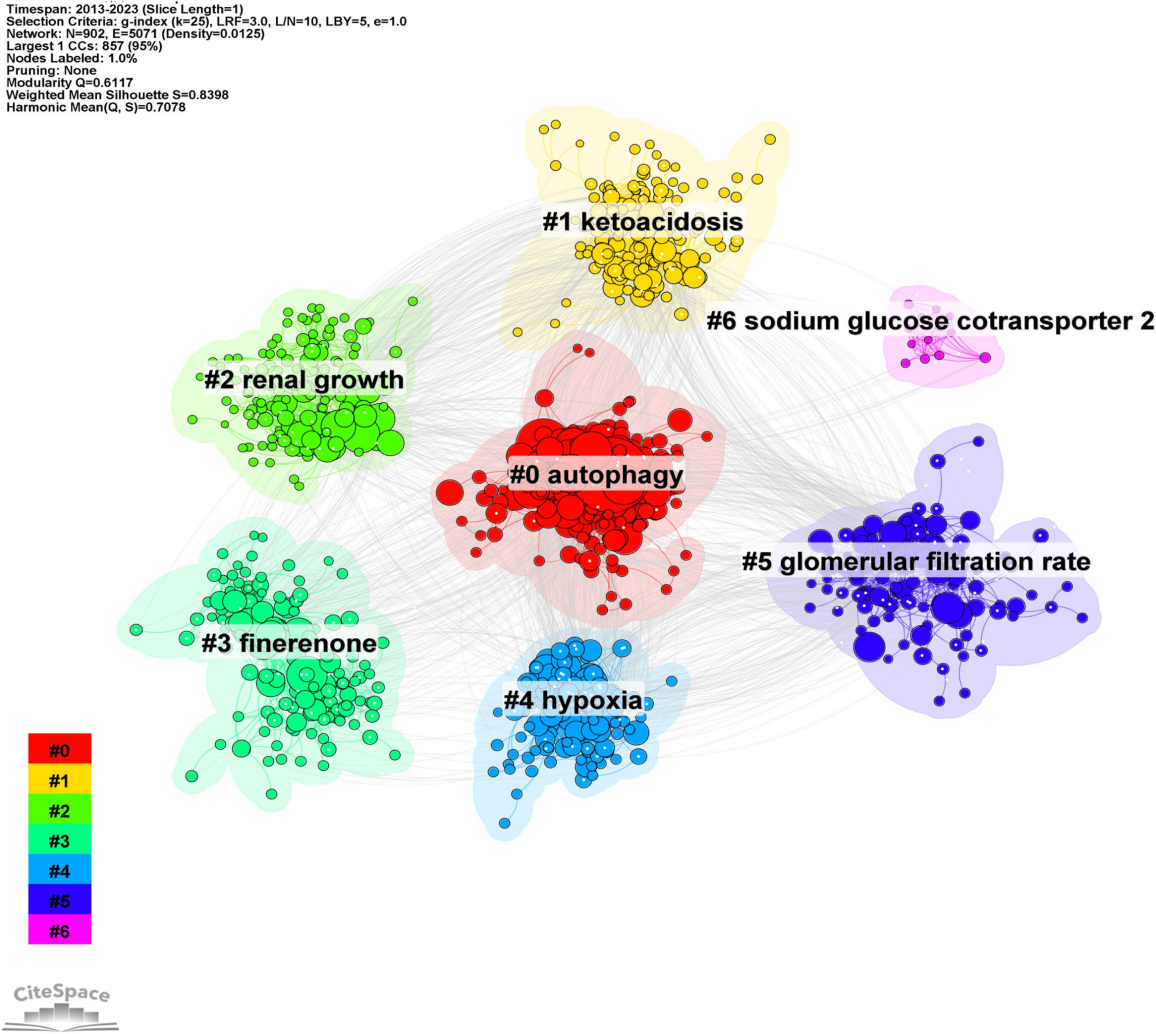
Citation clustering analysis of studies on SGLT2 inhibitors in the treatment of diabetic kidney disease.

We conducted a cluster analysis of references within the field of SGLT2 inhibitor research, as shown in Figure 6. Studies on SGLT2 inhibitors for diabetic kidney disease formed seven highly distinct clusters (modularity Q value = 0.6117), each exhibiting strong internal consistency (weighted average silhouette coefficient *S* = 0.8398). These clusters were labeled with terms such as ‘autophagy’, ‘ketoacidosis’, ‘SGLT2 inhibitors’, and ‘finerenone’. The distribution of these labels clearly indicates two major research foci: one centered on mechanisms of drug action (e.g., Cluster #6: sodium-glucose co-transporter 2 and Cluster #4: hypoxia) and the other on clinical treatment strategies (e.g., Cluster #3: finerenone and Cluster #5: glomerular filtration rate). This cluster analysis facilitates rapid identification of core research trajectories and emerging topics, providing researchers with a clear understanding of the forefront and primary directions in this area. For instance, the cluster focused on ‘empagliflozin’ highlights the crucial role this medication plays in treating diabetic kidney disease. Relevant clinical trials, such as the EMPA-REG OUTCOME study, provide significant evidence for the cardiovascular protective effects of SGLT2 inhibitors and spur extensive follow-up research [17]. Notably, ‘finerenone’ emerged as a distinct cluster, underscoring its prominent status in recent studies, particularly regarding combined therapy with SGLT2 inhibitors. Furthermore, this analysis provides a structured framework for exploring the mechanisms of cardiorenal protection [18]. The co-citation network facilitates the dissemination of these findings, enhancing the understanding of the multifaceted roles of SGLT2 inhibitors in managing diabetic kidney disease.

A co-citation analysis of the literature was conducted, yielding the following network analysis parameters: the number of nodes (N) is 902, and the number of edges (E) is 5071, resulting in a density of 0.0125. The largest connected component contains 857 nodes, representing 95% of the total. The modularity Q value is 0.6117, and the weighted average silhouette score (S) is 0.8398, yielding a harmonic mean of Q and S of 0.7078. The identified key themes among these clusters include ‘autophagy’, ‘ketoacidosis’, ‘SGLT2 inhibitors’, and ‘canagliflozin’, which underscore significant issues and research focal points in the realm of SGLT2 inhibitor studies.

### 3.6. Keyword co-occurrence clustering analysis

**Figure 7.**
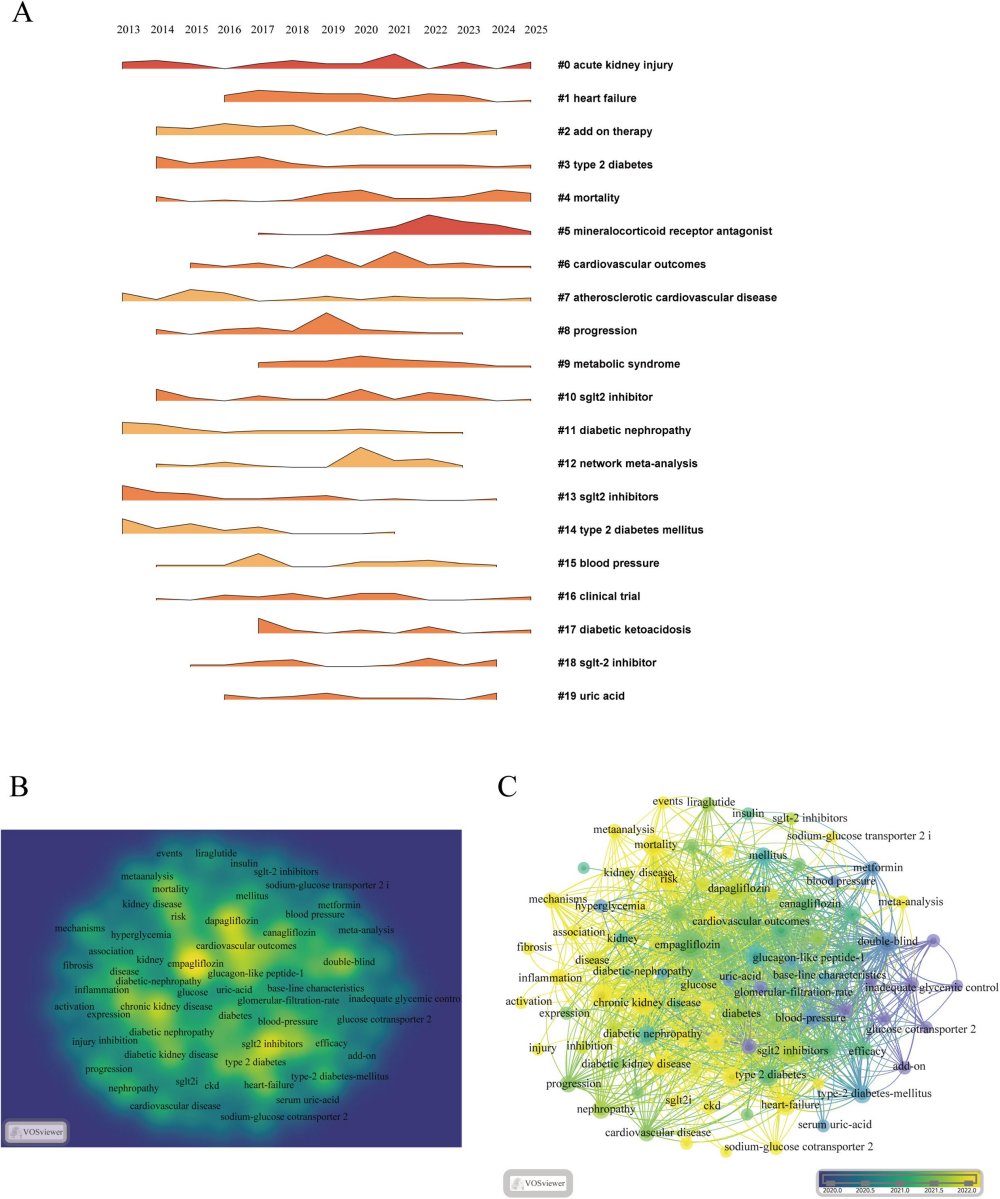
Keyword visualization analysis of research on SGLT2 inhibitors in the treatment of diabetic kidney disease. A. Keyword Co-occurrence Landscape Map: This visualization integrates chronological keyword evolution with cluster analysis to elucidate temporal trends in research themes. B. Keyword Co-occurrence Network: Node sizes and connection line thickness illustrate keyword frequency and complex interrelationships, highlighting core research themes and focal points. C. Keyword Cluster Timeline: Color-coded time periods display chronological keyword evolution, while color gradients visualize co-occurrence strength to reveal research hotspot clustering patterns.

To systematically analyze the evolving research themes over time, we conducted a temporal evolution and clustering analysis of keywords. Figure 7(A) illustrates the temporal trends in keyword density for various themes from 2013 to 2025. During the early phase (approximately 2013–2015), the most frequent keywords were ‘clinical trial’ (#16) and ‘SGLT2 inhibitors’ (#10), indicating that early research primarily focused on validating the basic efficacy of these drugs through clinical trial designs [19]. In the subsequent phase (approximately 2016–2018), there was a notable increase in the density of terms such as ‘type 2 diabetes’ (#3) and ‘blood pressure’ (#15). This shift marked a growing focus on the critical role of SGLT2 inhibitors in managing diabetic kidney disease and regulating metabolic parameters, including blood pressure. This change was largely driven by the pivotal EMPA-REG OUTCOME trial published in 2015, which confirmed the cardiovascular protective effects of empagliflozin in diabetic patients, thereby introducing the innovative concept of ‘cardiorenal protection’ into the forefront of research [20]. Notably, between 2019 and 2021, a significant surge occurred in the density of keywords such as ‘heart failure’ (#1), ‘cardiovascular outcomes’ (#6), ‘atherosclerotic cardiovascular disease’ (#7), and ‘mineralocorticoid receptor antagonist’ (#5). This increase closely correlates with groundbreaking advancements in the use of SGLT2 inhibitors for heart failure treatment, as evidenced by key trials like DAPA-HF [21]. In recent years (2022–2025), terms like ‘mineralocorticoid receptor antagonist’ (#5), ‘network meta-analysis’ (#12), and ‘mortality’ (#4) have gained prominence, reflecting an increased emphasis on evaluating the comparative effects of various interventions on long-term hard endpoints such as mortality and disease progression [22].

Figure 7(B) presents the clustering patterns of high-frequency keywords in a density word cloud format. The core area is densely populated with foundational terms like ‘SGLT2 inhibitors’ and ‘type 2 diabetes’, radiating outward to include keywords related to disease outcomes such as ‘heart failure’, ‘cardiovascular outcomes’, and ‘diabetic nephropathy’, as well as metabolic indicators like ‘uric acid’ and ‘blood pressure’. This hierarchical structure underscores a dual research focus, encompassing both the clinical effects of specific medications (e.g., the cardiovascular protective effects of empagliflozin) and the potential pathological mechanisms involved, such as the association between oxidative stress and renal injury. Additionally, the high co-occurrence of ‘cardiovascular outcomes’ and ‘chronic kidney disease’ highlights the emerging research trend of cardiorenal synergy in protection.

Figure 7(C) visualizes the research trajectory, dividing it into three collaboratively evolving phases through temporal clustering: the purple phase (approximately 2013–2018) is characterized by terms like ‘double-blind’, ‘uric acid’, ‘add-on therapy’, and ‘glomerular filtration rate’, reflecting an initial focus on preliminary efficacy validation and combination therapy strategies. The green phase (approximately 2019–2020) centers on ‘SGLT2 inhibitors’, ‘diabetic nephropathy’, and ‘type 2 diabetes’, indicating a deeper exploration of their roles in the management of diabetic kidney disease. The yellow phase (2021–2025) shows a strong concentration of terms such as ‘heart failure’, ‘cardiovascular outcomes’, ‘mortality’, ‘disease progression’, and ‘meta-analysis’, marking a shift toward evaluating their impacts on cardiovascular and renal hard endpoints while employing advanced statistical methods for comparative efficacy analysis.

This evolutionary trajectory delineates the developmental path of this field, beginning with early explorations of drug safety and preliminary disease applications, progressing to confirm mechanistic associations and benefits for cardiorenal outcomes, and ultimately focusing on optimizing long-term efficacy and comparative analyses of various treatment strategies.

### 3.7. Burst keyword analysis

**Figure 8.**
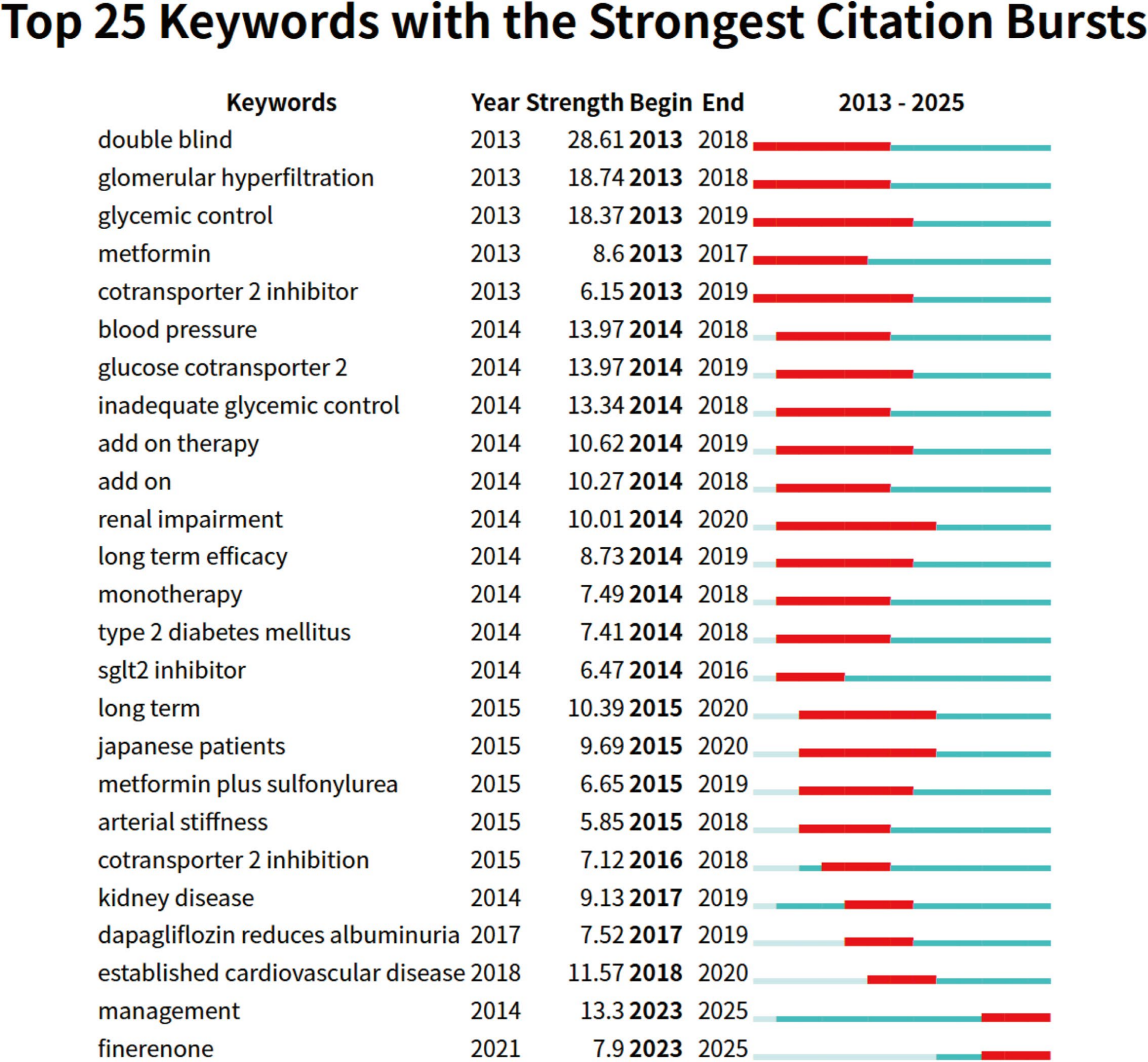
Top 25 representative burst keywords from highly cited references with strongest citation bursts. Keywords are arranged according to their initial burst year. The green timeline represents chronological segmentation, while the red segments highlight the specific start and end periods of each keyword’s burst. This figure demonstrates the dynamic trends of keywords from 2013 to 2025.

We employed CiteSpace’s burst detection algorithm to identify keywords with sudden citation frequency increases during specific periods, revealing chronological shifts in research focus. This algorithm effectively detects keywords showing significant growth trends, identifying emerging frontiers potentially missed by co-occurrence network analysis. Figure 8 presents the top 25 burst keywords by citation impact and frequency, along with their active periods from 2013 to 2025. These keywords reflect evolving research hotspots while indicating mainstream trends and cutting-edge directions.

During the initial research phase (2013–2015), keywords including ‘double-blind’ and ‘glomerular hyperfiltration’ emerged prominently, reflecting early emphasis on renal function improvement and rigorous trial design. The subsequent period (approximately 2014–2018) witnessed substantial broadening of research scope to encompass wider applications and key clinical endpoints of SGLT2 inhibitors. The concentrated emergence of keywords like ‘blood pressure’, ‘add-on therapy’, ‘monotherapy’, ‘type 2 diabetes’, ‘renal impairment’, and ‘long-term efficacy’ indicated a shift toward validating fundamental pharmacodynamics in type 2 diabetic kidney disease management and metabolic parameter effects. From approximately 2017, research focus narrowed to specific clinical benefits and particular patient populations. Keywords such as ‘kidney disease’, ‘dapagliflozin reducing albuminuria’, and ‘established cardiovascular disease’ marked intensified investigation into SGLT2 inhibitors’ role in delaying renal disease progression (evidenced by albuminuria reduction) and providing dual cardiorenal protection in diabetic patients with cardiovascular disease. In the most recent period (2023–2025), emerging keywords ‘management’ and ‘finerenone’ indicate current trends toward comprehensive disease management strategies and novel therapeutic agents exploration, particularly finerenone - a mineralocorticoid receptor antagonist frequently discussed alongside SGLT2 inhibitors.

Collectively, burst keyword evolution outlines the dynamic trajectory of SGLT2 inhibitor research focus. This progression evolved from initial pharmacodynamic validation and trial design toward assessing comprehensive benefits in complex clinical scenarios, particularly confirming cardiorenal protective effects. Ultimately, current focus advances toward refined disease management and novel treatment strategies. This evolutionary pathway reflects substantial field advancement from basic pharmacodynamic validation to clinical practice optimization and long-term patient outcome improvement.

## 4. Discussion

### 4.1. Research progress in SGLT2 inhibitors for diabetic kidney disease

This study systematically examined research progress on SGLT2 inhibitors in diabetic kidney disease (DKD) from 2013 to 2025. Global annual publication output grew exponentially, progressing from sporadic early reports to sustained high productivity in recent years. The United States has remained the leading contributor, supported by substantial research investment and the widespread conduct of multicenter clinical trials. Landmark studies, including EMPA-REG OUTCOME and DAPA-HF, provided a robust evidence base for the clinical application of SGLT2 inhibitors [23–25]. Institutional collaboration network analysis identified the University of Groningen and the University of Toronto as key hubs. By integrating metabolomics platforms, biobanks, and related resources, these institutions have accelerated mechanistic investigations [26]. The international collaboration network showed clear regional features and a tripolar structure comprising a United States-led blue cluster, a China-led green cluster, and a Germany-led red cluster. Notably, Japan ranked third worldwide in publication output (Table 1) but showed relatively limited engagement in international collaboration. This pattern may be related to language barriers or more locally oriented research strategies. These quantitative results not only map collaboration patterns but also imply potential barriers to knowledge diffusion. Future efforts should strengthen cross-regional collaboration, particularly with Asian regions that bear a high disease burden, to generate more broadly applicable evidence. Moreover, a large body of research has reached a consensus indicating that the public research funding system, exemplified by the National Institutes of Health, and the research and development investment from the pharmaceutical industry have been key drivers propelling large-scale clinical trials and translational research on SGLT2 inhibitors. This helps to explain the central position of the USA observed in this study, in terms of total publication output, high-impact research, and international collaboration networks [27].

**Table 1.** Top 10 journals publishing on SGLT2 inhibitors for diabetic kidney disease.

Rank	Productive journal	Number of publications	Citation	EISSN	JIF quartile	IF 2024
1	*Diabetes, Obesity & Metabolism*	163	5191	1463–1326	Q1	5.7
2	*International Journal of Molecular Sciences*	64	1669	1422–0067	Q1	4.9
3	*Journal of Clinical Medicine*	56	556	2077–0383	Q1	2.9
4	*Diabetes Therapy*	55	1332	1869–6961	Q3	2.6
5	*Nephrology Dialysis Transplantation*	51	1113	1460–2385	Q1	5.6
6	*Cardiovascular Diabetology*	50	1714	1475–2840	Q1	10.6
7	*Diabetes Care*	39	5286	1935–5548	Q1	16.6
8	*Frontiers in Endocrinology*	35	530	1664–2392	Q1	4.6
9	*Frontiers in Pharmacology*	34	422	1663–9812	Q1	4.8
10	*Diabetologia*	33	5223	1432–0428	Q1	10.2

Keyword co-occurrence mapping and burst detection confirmed a marked shift in research priorities. Early studies (approximately 2013–2015) primarily evaluated the fundamental pharmacodynamic effects of SGLT2 inhibitors and frequently adopted clinical trial designs such as the ‘double-blind trial’ [28]. After the publication of EMPA-REG OUTCOME and related findings, the mid period (2016–2021) increasingly focused on cardio-renal protective mechanisms and metabolic regulation [29–31]. This change represents more than a topic shift; it reflects a conceptual transition from viewing SGLT2 inhibitors as glucose-lowering agents to recognizing them as multi-organ protective therapies. In the most recent phase (2022–2025), research frontiers further concentrated on ‘mortality’, ‘management’, and ‘finerenone’. These trends indicate growing attention to long-term hard endpoints and more refined disease management, and they also highlight the emergence of combination therapy strategies. We critically assessed ‘finerenone’ as a burst keyword. Although trials such as FIDELIO-DKD reported additional renoprotective benefits when finerenone was combined with SGLT2 inhibitors, our bibliometric maps suggest persistent knowledge gaps. In particular, the molecular networks underpinning potential synergy (for example, interactions involving podocyte inflammation and tubular metabolic reprogramming) and the optimal use of combination strategies across different clinical phenotypes remain insufficiently characterized and should be priorities for future mechanistic studies [32].

Progress in this field has been driven by multiple factors, with several pivotal trials shaping both evidence and practice. The EMPA-REG OUTCOME trial (2015) first demonstrated cardiovascular benefits of empagliflozin and showed delayed kidney disease progression alongside reduced cardio-renal event risk, shifting attention toward ‘dual cardio-renal protection’ [33,34]. The pooled analysis of the CANVAS Program confirmed that canagliflozin reduced major adverse cardiovascular events (MACE) and provided kidney protection [35]. These results supported both cardiovascular and renal benefits and suggested a potential class effect of SGLT2 inhibitors. The DECLARE–TIMI 58 trial showed that dapagliflozin slowed kidney function decline in patients with type 2 diabetes and cardiovascular risk factors [36]. The DAPA-HF trial further demonstrated improved outcomes in patients with heart failure, independent of diabetes status [37]. Together, these studies helped shift the field from an exclusive focus on glycemic control to a broader multi-organ protection strategy.

We compared our findings with the bibliometric analysis by Zou and Sun (2019), which evaluated overall research in diabetic kidney disease from 2000 to 2017 [38]. Their study described a United States-led landscape, with early emphasis on renin–angiotensin system inhibition and a later shift toward podocytes, inflammation, and biomarkers. In contrast, our study extended the time window to 2013–2025 and focused specifically on SGLT2 inhibitors as a major therapeutic class. This focused scope allowed a more precise assessment of research dynamics and structural evolution within this subfield.

Earlier work established the leading role of the United States and highlighted traditional directions such as renin–angiotensin system inhibition and podocyte injury. Our analysis further shows that, with the expanding use of SGLT2 inhibitors, research priorities have shifted toward cardio-renal protective mechanisms and clinically oriented precision management. After the release of EMPA-REG OUTCOME results in 2015, our keyword evolution and clustering analyses identified ‘finerenone’ as an emerging frontier, reflecting the growing interest in combining SGLT2 inhibitors with mineralocorticoid receptor antagonists. This trend was not fully captured in earlier bibliometric studies. In journal distribution analysis, guided by Bradford’s law, we identified *Diabetes*, *Cardiovascular Diabetology*, and *Diabetes Care* as core journals in this field. Compared with earlier diabetic kidney disease research, core outlets have shifted from traditional nephrology journals (for example, *Nephrology Dialysis Transplantation*) toward journals centered on metabolism and cardiovascular disease. This change indicates that research on SGLT2 inhibitors is now deeply embedded in – and increasingly shapes – the integrated management paradigm for cardio-renal complications of diabetes, rather than remaining confined to nephrology alone.

### 4.2. Research hotspots and emerging trends of SGLT2 inhibitors in diabetic kidney disease

Keyword burst analysis clearly delineated the stage-specific evolution of research hotspots related to SGLT2 inhibitors in diabetic kidney disease. In the early phase (2013–2015), ‘glomerular hyperfiltration’ emerged as the dominant focus, consistent with the renal hemodynamic actions of SGLT2 inhibitors [39]. Mechanistic evidence indicates that these agents relieve intraglomerular hypertension by inhibiting sodium–glucose reabsorption in the renal proximal tubule and thereby activating tubuloglomerular feedback. This core mechanism provides a strong theoretical basis for the subsequent recognition of their renoprotective effects [40]. Meanwhile, the pronounced burst of ‘double-blind trial’ reflects the consolidation of evidence-based standards in this research area. Such rigorous trial design principles underpinned pivotal studies, including EMPA-REG OUTCOME [41].

The mid-stage evolution (2014–2020) showed a clear broadening of research directions. The sustained burst of ‘blood pressure control’ supports the osmotic diuretic effects of SGLT2 inhibitors on systemic hemodynamics and their capacity to enhance uric acid excretion [42]. In parallel, the persistent burst of ‘kidney injury’ (2014–2020) indicates intensified investigation into safety and dose-adjustment strategies for patients with impaired kidney function, which contributed to updates in clinical guidelines [43]. Importantly, the concurrent emergence of ‘long-term efficacy’ and ‘add-on therapy’ marked a pivotal shift in clinical emphasis. Research moved from evaluating single-agent effects to clarifying mechanisms and outcomes of combination regimens [44]. For instance, metformin combined with SGLT2 inhibitors can improve insulin sensitivity through complementary actions, namely AMP-activated protein kinase activation and SGLT2 inhibition. This complementarity helps explain why this regimen is consistently recommended as a first-line combination in clinical guidelines [45].

Recent advances (2017–2023) centered on organ-protective mechanisms. The burst term ‘dapagliflozin reduces proteinuria’ aligns with the key findings of the DAPA-CKD trial, in which dapagliflozin reduced proteinuria by 40% [46]. Subsequent studies suggest that this reduction is independent of glucose lowering. As indicated by the ‘podocyte injury’ node in the accompanying figure, a plausible molecular explanation is that inhibition of glucose transport attenuates inflammatory signaling, including reduced tumor necrosis factor-α (TNF-α) release [47]. In addition, the strong burst of ‘cardiovascular disease’ reflects mechanisms highlighted by EMPA-REG OUTCOME. In that trial, SGLT2 inhibitors were associated with improved myocardial energy utilization (for example, increased ketone use) and a 38% reduction in cardiovascular death [48].

The current frontier (2023–2025) is characterized by ‘integrated management’ and ‘finerenone’. This pattern represents a higher-level shift in therapeutic strategy, moving from single-target intervention to multi-pathway blockade [49]. The FIDELIO-DKD trial reported that finerenone combined with SGLT2 inhibitors can provide dual pathway modulation involving the renin–angiotensin–aldosterone system (RAAS) and metabolic mechanisms, further reducing the risk of composite kidney outcomes by 31% [50]. This apparent synergy may arise because mineralocorticoid receptor antagonists suppress renal fibrotic progression *via* the transforming growth factor-β1 (TGF-β1)/Smad3 pathway, whereas SGLT2 inhibitors primarily reduce intraglomerular hypertension [51]. However, bibliometric mapping indicates that mechanistic evidence remains limited on how these drug classes interact at the cellular level – such as within podocytes, mesangial cells, and tubular epithelial cells – to coordinate anti-fibrotic and anti-inflammatory effects. Addressing this gap should be a priority for future mechanistic research.

### 4.3. Future research directions based on bibliometric analysis

Through quantitative and visualization-based analyses of the literature, this study not only delineated the field’s macroscopic knowledge structure but also identified several notable gaps in current research. First, research collaboration across regions remains uneven. In particular, both intra- and interregional collaboration density appears relatively limited in Asia, a high-burden region, which may restrict the generation of region-specific evidence. Second, with respect to mechanisms, although keyword analysis and co-citation clustering highlighted themes such as cardio-renal protection and combination therapy, the literature provides limited mechanistic depth. Specifically, evidence remains insufficient on synergistic interaction networks and on cell type–specific effects across distinct kidney cell populations. Third, the translational pathway from research hotspots to clinical practice is not well defined. A key limitation is the lack of clear guidance on how to tailor treatment to heterogeneous patient profiles and how to adopt outcome measures that better reflect routine clinical care. In this context, our study offers three main contributions. First, we mapped a three-stage trajectory in which research on SGLT2 inhibitors evolved from basic pharmacodynamic studies to integrated cardio-renal protection and then toward precision-oriented management. Second, using advanced algorithms, we identified emerging directions such as ‘finerenone combination therapy’, providing additional perspectives for future work. Third, we characterized regional differences in collaboration patterns, offering evidence that may help optimize global research partnerships and resource allocation. Together, these findings update the knowledge map of this field and inform future research planning and strategic prioritization.

Based on these results, we conducted multidimensional cross-validation by integrating the sustained influence of high-centrality keywords, the thematic evolution of clusters derived from highly cited references, and the research emphasis of recently highly cited articles. We therefore propose the following targeted directions for future research: First, to address the imbalance observed in collaboration networks, future work should prioritize multinational prospective cohorts and multicenter clinical trials in Asia. These studies should assess the long-term efficacy of SGLT2 inhibitors in Asian populations and include region-relevant endpoints, such as morning blood pressure surge and stroke risk. Second, to strengthen mechanistic understanding, future studies should apply emerging approaches such as single-cell transcriptomics and spatial metabolomics. Priority should be given to defining coordinated regulatory networks through which SGLT2 inhibitors and mineralocorticoid receptor antagonists modulate podocyte inflammasome activity and tubular metabolic reprogramming, with particular attention to complementary pathways that suppress renal fibrosis [52]. Third, research should extend toward real-world evidence and more refined clinical decision-making. Future studies should move beyond the hard-endpoint framework of classical randomized trials to validate efficacy and safety in real-world datasets across key subgroups (for example, older adults and individuals with advanced chronic kidney disease). In parallel, biomarker- or phenotype-guided individualized treatment strategies should be developed and evaluated [53].

In summary, based on this bibliometric analysis, we anticipate that future research should prioritize three areas. First, international collaboration should be strengthened, with particular emphasis on expanding partnerships within Asia. Second, multi-omics approaches should be integrated to clarify synergistic mechanisms between SGLT2 inhibitors and mineralocorticoid receptor antagonists (such as finerenone) and to identify drug-responsive targets across different kidney cell types. Third, clinical trial designs should be optimized to incorporate region-specific endpoints, including stroke risk and morning blood pressure surge, thereby improving translational impact and clinical relevance.

### 4.4. Strengths and limitations

Bibliometrics is a systematic research approach with clear strengths. It can process large volumes of literature efficiently and, when combined with data-mining and visualization tools, can identify knowledge structures, emerging trends, and landmark publications. In this study, we analyzed 2,245 publications. This analysis not only outlined the field’s broad developmental trajectory but also sought to generate actionable insights through clinically informed interpretation of quantitative findings. For example, we evaluated the potential and limitations of finerenone-based combination therapy from a clinical perspective to provide perspectives that may inform future research.

Nevertheless, bibliometric analysis has inherent limitations. First, because we used only the Web of Science Core Collection, relevant studies may have been missed, particularly those published in regional journals not indexed in this database. Second, citation lag may lead to underrepresentation of recently published high-quality studies and may overemphasize highly cited topics, which can affect the interpretation of the latest research trends. Third, we included only English-language publications, which may have excluded important findings reported in other languages and reduced the overall completeness of the evidence base. In addition, we set a minimum threshold of 15 publications per author. This choice may have underestimated contributions from lower-frequency authors. Future studies could incorporate complementary indicators, such as citation impact and collaboration breadth, to evaluate author influence more comprehensively. Finally, purely quantitative results can be descriptive and may not fully capture mechanistic or clinical meaning. To address this issue, we strengthened qualitative assessment and clinical interpretation of the bibliometric outputs. For instance, we linked burst keywords to mechanistic evidence from pivotal clinical trials to reduce reliance on numerical patterns alone. Despite these constraints, critical interpretation of bibliometric maps within a domain-knowledge framework still allowed us to delineate major developmental patterns and research hotspots related to SGLT2 inhibitors in diabetic kidney disease research. Future work could mitigate these limitations by integrating multiple databases, combining qualitative and quantitative methods, including multilingual literature, and refining analytical techniques. Such an integrated strategy would provide stronger evidence to support ongoing research on SGLT2 inhibitors in diabetic kidney disease.

## 5. Conclusion

This bibliometric analysis elucidates the research landscape and evolving trends of SGLT2 inhibitors in diabetic kidney disease. The results demonstrate a marked concentration of research contributions, with the United States, the University of Groningen, and lead researcher Heerspink H.J.L. emerging as the most influential entities. Analysis of temporal patterns reveals a clear transition in research priorities: initial studies focused on establishing fundamental efficacy and safety, later shifting toward cardiorenal protective mechanisms and clinical outcomes, and more recently advancing toward personalized management strategies and innovative combination therapies such as with finerenone. Building on these insights, three key directions are proposed for future research. First, enhancing international collaboration is essential to foster cross-regional and interdisciplinary synergy. Second, applying multi-omics technologies will help clarify the synergistic mechanisms between SGLT2 inhibitors and other agents like mineralocorticoid receptor antagonists. Finally, clinical trials should adopt more representative endpoints – such as stroke risk and morning blood pressure surge – to improve the generalizability and translational impact of study results.

## Supplementary Material

Lay summary.doc

Supplementary materials.doc

## Data Availability

The data (analytical dataset) supporting the findings of this study are available from the corresponding author upon reasonable request. The bibliographic data used for this study are publicly available through the Web of Science Core Collection (Clarivate Analytics), access to which may require an institutional subscription.
