# Supplementary material for: Bibliometric analysis of SGLT2 inhibitor treatment for diabetic kidney disease
Source: Ren Fail. 2026 May 21;48(1):2667611. doi: 10.1080/0886022X.2026.2667611 (PMC13195706; doi:10.1080/0886022X.2026.2667611)
Supplement: Lay summary.doc [file IRNF_A_2667611_SM5516.doc]

**Lay (plain language) summary**
**Manuscript ID:**  LRNF-2025-SR-0660.R1

**Manuscript number：**255417274

**Title：**Bibliometric Analysis of SGLT2 Inhibitor Treatment for Diabetic Kidney Disease

 This study adopted a big data bibliometric approach to systematically summarize 2,415 high-quality studies on sodium-glucose co-transporter 2 (SGLT2) inhibitors for the treatment of diabetic kidney disease published from 2013 to 2025, and fully presented the developmental context and core findings of this field. From a global perspective, the annual number of publications on relevant research has shown an exponential growth trend. The United States, China and England are the core contributing countries, and the University of Groningen together with the core scholar teams have formed the most influential research network in this field. Through co-citation and keyword analysis, we divided the research course into three stages: the early stage (2013-2015) focused on clinical trial design to verify the basic glucose-lowering and renoprotective efficacy of SGLT2 inhibitors; the middle stage (2016-2021) shifted to the research on cardiorenal protective mechanisms, with a focus on exploring pathological pathways such as autophagy and hypoxia to clarify the multi-organ protective effects of the drugs; the recent and future stage (2022-2025) has turned the research focus to the combination therapy of SGLT2 inhibitors and finerenone, fine disease management and hard endpoint verification, while emphasizing the importance of international collaboration and region-specific research. Future research needs to further deepen the study on the mechanisms of combination therapy and design clinical trials that are more suitable for patients in different regions, so as to promote the individualization and precision of the treatment for diabetic kidney disease.
