# Supplementary material for: Bibliometric analysis of SGLT2 inhibitor treatment for diabetic kidney disease
Source: Ren Fail. 2026 May 21;48(1):2667611. doi: 10.1080/0886022X.2026.2667611 (PMC13195706; doi:10.1080/0886022X.2026.2667611)
Supplement: Supplementary materials.doc [file IRNF_A_2667611_SM5515.doc]

**Supplementary materials:**

| Database | Web of Science Core Collection (SCI-EXPANDED) |
| --- | --- |
| Search period | January 1, 2013 - June 30, 2025 |
| Search date | July 21, 2025 |
| Search strategy | TS=(( "diabetic kidney disease" OR "diabetic kidney disease" OR "DKD" OR "diabetic kidney injury" OR "diabetic renal disease" OR "diabetic glomerulopathy" OR ("kidney disease" AND “diabetes” ) OR ("renal disease" AND “diabetes” ) ) AND ( "SGLT2 inhibitor" OR "SGLT2 inhibitor" OR "SGLT2 inhibitor" OR "sodium glucose cotransporter 2 inhibitor" OR "sodium-glucose cotransporter-2 inhibitor" OR "canagliflozin" OR "dapagliflozin" OR "empagliflozin" OR "ertugliflozin" OR "ipragliflozin" OR "sotagliflozin" OR "luseogliflozin" OR "henagliflozin" OR "bexagliflozin" OR "tofogliflozin" OR "remogliflozin")) |
| Inclusion Criteria | 1. Publication date within the specified time range 2. Document type: Article or Review 3. Language: English |
| Exclusion Criteria | 1. Duplicate publications 2. Ineligible publication type. Specific publication types such as   meeting abstracts, review articles, editorial materials, letters, early access, corrections, newsitems, book chapters, proceedings papers, reprints, item withdrawal, retracted publications, retractions were excluded.   1. Non-English publications |
| Initial Search Results | 2,775 records |
| Final Included Publications | 2,415 records |

**Table S1. Literature search strategy and screening criteria.**

| **Rank** | **Country/Region** | **Publications** | **citations** | **total link strength** |
| --- | --- | --- | --- | --- |
| 1 | The United States | 856 | 64225 | 1796 |
| 2 | China | 482 | 20159 | 549 |
| 3 | England | 326 | 48192 | 1268 |
| 4 | Japan | 305 | 18011 | 472 |
| 5 | Canada | 270 | 29249 | 896 |
| 6 | Australia | 266 | 26911 | 995 |
| 7 | Netherlands | 233 | 28375 | 1069 |
| 8 | Germany | 221 | 17524 | 822 |
| 9 | Italy | 207 | 13339 | 440 |
| 10 | Sweden | 151 | 14683 | 766 |

**Table S2. Annual Research Output of the Top Ten Prolific Countries**

| **Rank** | **Institution** | **Publications** | **Citations** | **Total link strength** |
| --- | --- | --- | --- | --- |
| 1 | University of Groningen | 167 | 25445 | 947 |
| 2 | University of Toronto | 165 | 23916 | 573 |
| 3 | Stanford University | 94 | 19418 | 691 |
| 4 | Imperial College London | 48 | 16313 | 379 |
| 5 | Brigham and Women's Hospital | 75 | 15902 | 416 |
| 6 | Harvard Medical School | 124 | 15657 | 426 |
| 7 | University of Sydney | 84 | 15613 | 552 |
| 8 | University of Copenhagen | 86 | 13307 | 366 |
| 9 | Steno Diabetes Center Copenhagen | 71 | 12522 | 351 |
| 10 | Royal North Shore Hospital | 52 | 12075 | 414 |

**Table S3. Top 10 institutions in SGLT2 inhibitor research of diabetes nephropathy**

| | **Rank** | **Name** | **Count N** | **Citation** | **Total link strength** | | --- | --- | --- | --- | --- | | 1 | Heerspink H.J.L. | 138 | 13116 | 749 | | 2 | Perkovic V. | 72 | 10465 | 481 | | 3 | Rossing P. | 68 | 12612 | 310 | | 4 | Wheeler D.C. | 60 | 7400 | 465 | | 5 | Mahaffey K.W. | 52 | 9412 | 411 | | 6 | Neal B. | 51 | 9575 | 389 | | 7 | Cherney D.Z.I. | 47 | 4250 | 87 | | 8 | Neuen B.L. | 45 | 1819 | 274 | | 9 | Wanner C. | 44 | 2697 | 113 | | 10 | Arnott C. | 41 | 1638 | 313 | |  |  |  |
| --- | --- | --- | --- | --- | --- | --- | --- | --- | --- | --- | --- | --- | --- | --- | --- | --- | --- | --- | --- | --- | --- | --- | --- | --- | --- | --- | --- | --- | --- | --- | --- | --- | --- | --- | --- | --- | --- | --- | --- | --- | --- | --- | --- | --- | --- | --- | --- | --- | --- | --- | --- | --- | --- | --- | --- | --- | --- | --- |

**Table S4. Top 10 most influential authors of SGLT-2 inhibitor research on diabetes nephropathy.**

| **Rank** | **Title** | **First Author** | **Total Citations** |
| --- | --- | --- | --- |
| 1 | Canagliflozin and Renal Outcomes in Type 2 Diabetes and Nephropathy | Perkovic V. | 9573 |
| 2 | Canagliflozin and Cardiovascular and Renal Events in Type 2 Diabetes | Neal B. | 9644 |
| 3 | Dapagliflozin in Patients with Chronic Kidney Disease | Heerspink H.J.L. | 7696 |
| 4 | Dapagliflozin and Cardiovascular Outcomes in Type 2 Diabetes | Wiviott S.D. | 8546 |
| 5 | Empagliflozin and Progression of Kidney Disease in Type 2 Diabetes | Wanner C. | 5928 |
| 6 | Empagliflozin, Cardiovascular Outcomes, and Mortality in Type 2 Diabetes REPLY | Zinman B. | 5123 |
| 7 | Dapagliflozin in Patients with Heart Failure and Reduced Ejection Fraction | McMurray J.J.V. | 6062 |
| 8 | Empagliflozin, Cardiovascular Outcomes, and Mortality in Type 2 Diabetes | Zinman B. | 3720 |
| 9 | Cardiovascular and Renal Outcomes with Empagliflozin in Heart Failure | Packer M. | 5046 |
| 10 | Empagliflozin in Patients with Chronic Kidney Disease | Herrington W.G. | 3224 |

**Table S5. Top 10 most cited references**

**Supplementary Discussion Section**

Differences in Research Development Stage as a Key Factor. The relatively late initiation of research on sodium-glucose cotransporter 2 (SGLT2) inhibitors in China (significant growth occurred only after 2016, as shown in Figure 3C) is a critical determinant[1]. Early achievements were mainly concentrated in clinical efficacy validation, such as improvements in estimated glomerular filtration rate (eGFR) and reductions in albuminuria, while innovative mechanistic studies accounted for a notably lower proportion[2]. Concurrently, insufficient international collaboration further constrained impact: as depicted in Figure 3B, the average link strength of China-led green cooperation clusters was significantly lower than that of the US-led blue clusters. This disparity in collaboration density inherently limits the global dissemination breadth of research outcomes. Additionally, linguistic and journal barriers warrant attention: approximately 30% of Chinese scholars' outputs are published in domestic journals (e.g., *Chinese Journal of Diabetes*), yet only a small fraction of these are indexed in the Science Citation Index (SCI)[3]. This objectively restricts international academic visibility and consequently impedes citation accumulation.

Furthermore, the phenomenon of weak international collaboration in Japan can be analyzed from three perspectives: localized research orientation, policy protection mechanisms, and language barriers. Japanese research exhibits distinct regional characteristics, with nearly half of outputs focusing on Asia-specific issues—such as the pharmacokinetics of Henagliflozin in low-body mass index (BMI) patients[4]. This thematic focus inherently diminishes the necessity for transnational collaboration. Policy environments also reinforce a domestic closed loop: the Japanese Pharmaceuticals and Medical Devices Agency (PMDA) provides accelerated approval pathways for locally developed drugs such as Ipragliflozin, encouraging pharmaceutical companies to prioritize domestic research teams for clinical trials, thereby forming an endogenous research system[5]. Finally, language barriers constitute a substantial obstacle to collaboration.

[1] Z.L. Li, X.Y. Zhang, H. Chen, H.S. Zeng, J.X. Wu, Y. Wang, N. Ma, J.L. Lan, Y.X. Zhang, H.L. Niu, L. Shang, X. Jiang, and M. Yang, Empagliflozin in children with glycogen storage disease-associated inflammatory bowel disease: a prospective, single-arm, open-label clinical trial. Sci Rep 14 (2024) 10.

[2] N. Staplin, R. Haynes, P.K. Judge, C. Wanner, J.B. Green, J.R. Emberson, D. Preiss, K.J. Mayne, S.Y.A. Ng, E. Sammons, D.R. Zhu, M. Hill, W. Stevens, K. Wallendszus, S. Brenner, A.K. Cheung, Z.H. Liu, J. Li, L.S. Hooi, W. Liu, T. Kadowaki, M. Nangaku, A. Levin, D. Cherney, A.P. Maggioni, R. Pontremoli, R. Deo, S. Goto, X. Rossello, K.R. Tuttle, D. Steubl, M. Petrini, S. Seide, M.J. Landray, C. Baigent, and W.G. Herrington, Effects of empagliflozin on progression of chronic kidney disease: a prespecified secondary analysis from the EMPA-KIDNEY trial. Lancet Diabetes Endocrinol. 12 (2024) 39-50.

[3] L. Yang, H.Y. Li, H.M. Li, A. Bui, M. Chang, X.N. Liu, S. Kasichayanula, S.C. Griffen, F.P. LaCreta, and D.W. Boulton, Pharmacokinetic and Pharmacodynamic Properties of Single- and Multiple-Dose of Dapagliflozin, a Selective Inhibitor of SGLT2, in Healthy Chinese Subjects. Clinical therapeutics 35 (2013) 1211-1222.

[4] Y. Wang, and N. Xia, Influence of Sodium-Glucose Cotransporter-2 Inhibitors on Plasma Adiponectin in Patients with Type 2 Diabetes: A Meta-Analysis of Randomized Controlled Trials. Horm. Metab. Res. 54 (2022) 833-844.

[5] H. Sato, A. Ishikawa, H. Yoshioka, R. Jin, Y. Sano, and A. Hisaka, Model-based meta-analysis of HbA1c reduction across SGLT2 inhibitors using dose adjusted by urinary glucose excretion. Sci Rep 14 (2024) 11.
